# Evaluation of stability of three different mini-implants, based on thread shape factor and numerical analysis of stress around mini-implants with different insertion angle, with relation to en-masse retraction force

**DOI:** 10.1590/2177-6709.25.6.059-068.oar

**Published:** 2020

**Authors:** Safiya Sana, Rekha Reddy, Ashok Kumar Talapaneni, Arshad Hussain, Sayeeda Laegue Bangi, Asma Fatima

**Affiliations:** 1 Al-Badar Rural Dental College & Hospital, Department of Orthodontics & Dentofacial Orthopaedics (Gulbarga/KA, India).

**Keywords:** Thread shape factor, Primary stability, FEM

## Abstract

**Objectives:**

Assess the stability of three different mini-implants, based on thread shape factor (TSF), and evaluate stresses at the mini-implant site and surrounding cortical bone on application of retraction force, at two different insertion angles.

**Methods:**

Mini-implants of three different diameters (M1 - Orthoimplant, 1.8mm), (M2 - Tomas, 1.6mm) and (M3 - Vector TAS, 1.4mm) and length of 8mm were used. Using scanning electronic microscopy, the mean thread depth, pitch and relationship between the two (TSF) were calculated. The mini-implants were loaded into a synthetic bone block and the pull-out strength was tested. One way ANOVA and Tukey *post-hoc* tests were used to compare the pull-out strength of mini-implants. P values < 0.05 were considered statistically significant. Finite element models (FEM) were constructed with insertion angulation at 90° and 60°, with retraction force of 150 g. The results were analyzed using ANSYS software.

**Results:**

Statistically significant difference was found among all the three mini-implants for thread depth and pitch (< 0.001). Statistically significant higher pull-out force value was seen for Orthoimplant. The stress distribution level in mini-implant and surrounding bone was observed to be smaller for Orthoimplant.

**Conclusion:**

Orthoimplant mini-implants have more favorable geometric characteristics among the three types, and less stress with 90°angulation.

## RESUMO

**Objetivos:**

Avaliar a estabilidade de três diferentes tipos de mini-implantes, com base no fator formato da rosca (*thread shape factor,* TSF), e avaliar a tensão no local de inserção e no osso cortical ao redor dos mini-implantes inseridos com dois ângulos diferentes, durante a aplicação de força para retração.

**Métodos:**

Foram usados três diferentes diâmetros de mini-implantes, sendo eles 1,8 mm (M1, ORTHO Implant), 1,6 mm (M2, Tomas) e 1,4 mm (M3, Vector TAS), todos com comprimento de 8 mm. Por meio da microscopia eletrônica de varredura, foram calculados a profundidade da rosca, o passo da rosca (distância entre os filetes da rosca) e a relação entre os dois (TSF). Para realização do teste de tração (*pull-out*), os mini-implantes foram inseridos em um bloco de osso sintético. Os testes ANOVA de uma via e *post-hoc* de Tukey foram usados para comparar as forças de resistência à tração dos mini-implantes, considerando-se estatisticamente significativos valores de *p*< 0,05. Modelos de elementos finitos (MEF) foram gerados com ângulos de inserção dos mini-implantes a 90° e 60°, com força de retração em 150g. Os resultados foram analisados usando-se o *software* ANSYS.

**Resultados:**

Diferenças estatisticamente significativas foram encontradas entre os três mini-implantes quanto à profundidade da rosca e o passo da rosca (*p*< 0,001). O ORTHO Implant apresentou a maior força de resistência à tração, com significância estatística. O nível de distribuição das tensões no mini-implante e no osso circundante também foi menor para o ORTHO Implant.

**Resultados:**

Entre os diferentes tipos de mini-implantes analisados, o ORTHO Implant apresentou as características geométricas mais favoráveis e a menor tensão com o ângulo de inserção de 90°.

## INTRODUCTION

The need for orthodontic treatment modalities that maximize anchorage control and minimize patient compliance has led to the development of mini-implant-assisted orthodontics.^1^ Temporary anchorage devices (TADs) in the form of mini-implants are used as a skeletal anchorage and their utilization has become a reliable and acceptable method.^2^

Primary stability of mini-implant is due to the mechanical interlock between the bone and mini-implant, and it depends on many factors, including bone quality, mini-implant site and insertion angle, and design of mini-implants, such as diameter, thread form, pitch, thread size, mini-implant material,^3^ ^-^ ^5^ and the recently introduced thread shape factor (TSF)^2^. TSF is calculated as the geometrical relationship between the mean thread depth and the pitch (D/P) and is expressed as a percentage.^2^

Bone remodeling processes at the bone/screw interface are correlated with the structural response of the bony tissue to the TADs and then to the stress/strain field, developing within themselves and the surrounding bone.^6^ Studies of stress allow optimization of the shape and geometric parameters. A key to the success or failure of mini-implant is the manner in which stresses are transferred to the surrounding bone.^7^

The proper insertion angle is important for cortical anchorage, patient safety (root damage), and biomechanical control. It also provides increased surface contact area between the mini-implant and the bone.^8^

Measurement of the stresses *in vivo* is virtually impossible. The finite element method (FEM) is thus a valid technique used to analyze structural stress.^9^ In order to understand better how a viscous-elastic material, such as the bone (cortical and cancellous layer), reacts to the insertion of rigid material like titanium, and which kind of stress can be generated by a specific thread design, FEM analysis can be utilized to serve this purpose.^2^

However, the literature lacks information on the combination of ideal geometric design characteristics, i.e., TSF and optimal insertion angle during *en-masse* retraction. To address that, this study was conducted to evaluate the effect of TSF of 3 different mini-implants, and their various insertion angle combinations, on the pull-out strength and stresses at the mini-implant site and surrounding bone during *en-masse* retraction, using a FEM study.

## MATERIAL AND METHODS

Detailed geometry of all three mini-implants was studied through scanning electron microscope (SEM), to measure the TSF.

Pull-out test was carried out to determine the primary stability.

FEM was done to evaluate stress distribution at the mini-implant site and in the surrounding cortical bone, with the application of retraction force at two different insertion angles (60° and 90°).

### Material

The three mini-implants used in the study were as follows:

1) ORTHOImplant (3M Unitek, Monrovia, CA, USA): 1.8-mm diameter and 8-mm length (M1).
2) TOMAS (Dentaurum): 1.6-mm diameter and 8-mm length (M2).
3) VECTOR TAS (Ormco): 1.4-mm diameter and 8-mm length (M3).

According to the manufacturer’s description, these mini-implants are available in the above mentioned diameter with three different lengths. All mini-implants are made of Ti-6Al-4V alloy.

For the pull-out test, double layer artificial bone block (Sawbones, Pacific Research Laboratories Inc, Vashon, Washington;) was used. The block is composed by a polyurethane foam, measuring 120 x 170 x 41 mm thick, having a 1.1-mm top layer with a 40-pcf density, and a 39-mm base layer with a 10-pcf density (Table 1).^10^

**Table 1:** Material properties of artificial bone materials (poisson ratio = 0.3).

	Strength and modulus (MPA)					
	Compressive		Tensile		Shear	
Density pcf (g/cc)	Strength	Modulus	Strength	Modulus	Strength	Modulus
10 (0.16)	22	58	2.1	86	1.6	19
40 (0.64)	31	759	19	1000	11	130

### Methods

#### Scanning electron microscopy

**Figure 1:**
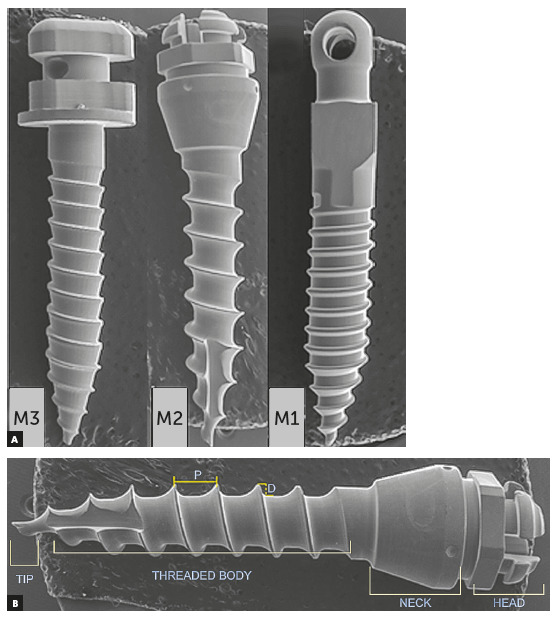
A) M1, M2, M3: SEM images of mini-implants at magnification x27. B) Illustrating parts of mini-implant (D is thread depth and P is pitch).

**Figure 2:**
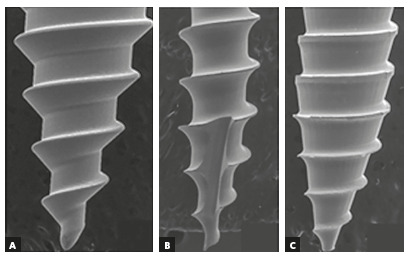
SEM images of mini-implants at magnification x 33.

Each mini-implant was examined using a scanning electron microscope (Tescan Vega3, Czech Republic), operating at 30.00 kV, which was performed at Sanray laboratories Pvt Ltd (Hyderabad, India). Images of each mini-implant were captured with VEGA 3.0 software, and obtained at 27× and 33× magnifications (Figs 1 and 2). The pitch and thread depth was measured using team measurement tool of Biovis Materials VA4.59 software.

#### Pull-out test

Pull-out strength tests were performed at the same laboratory on bone blocks constructed featuring a superficial layer with biomechanical characteristics (elasticity, hardness and density) similar to the cortical bone and a deeper layer with characteristics mimicking the trabecular bone. Computed tomography study by Migliorati et al.^13^ reported a mean cortical thickness of 1.10mm on the buccal side of the maxilla. So a bone block with 1.1-mm cortical thickness was used in the present study.

This bone block was divided into small blocks measuring 1.5cm x 2.5cm, so that it could fit accurately in between the metal plates of the testing machine. The geometric center was marked on each bone block and the mini-implants were inserted at these points, to a thread depth of 6mm. The pull-out test was carried out by a universal testing machine Shimadzu AGS-X featuring 5 kN load. The mini-implant was loaded with a traction speed of 2mm/minute and the pull-out strength was measured as the peak force recorded by the built-in machine software (Trapezium v. 1.4.5). The method was repeated for each mini-implant.

#### Finite element method

**Figure 3:**
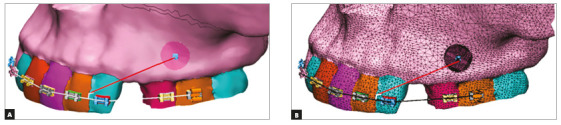
Three-dimensional geometric model of half maxilla with brackets, mini-implant, archwire with crimpable hook and NiTi closed coil spring.

For creating a finite element model, a 3D CAD model was constructed from a CT scan of the craniofacial complex of a 15-years-old female patient. CT scan images of the maxillary bone were taken by Siemens Somatom Definition 64 (120kVp; 290 mAs) in axial direction. Sequential CT images were taken at 0.5 mm intervals to reproduce finer and detailed aspects of the geometry. A total of 625 images were stacked over one another and converted to a finite element meshed model by the software MIMIC (version 18.0). Tetrahydron elements were used to mesh the skull and teeth. Archwire, brackets, crimpable hooks and NiTi closed coil spring were modeled by the software ANSYS Design Modeler (version 19; ANSYS Inc., Integrated Design Analysis Consultants, INDIA Pvt Ltd) with beam elements. The total number of elements in the geometry was 864,650 and the total number of nodes created was 247,119 (Fig 3). Nodes and elements defined for each model of mini-implants (M1, M2 and M3) for 90^o^ and 60^o^ angulations, respectively, is presented in Table 3.

Only one side of the maxilla was generated, as results on the other side are expected to be the same. To simulate the extraction space, maxillary first premolar was removed from the model. Geometric model of brackets, mini-implants, archwire with crimpable hook and nickel-titanium closed coil spring were constructed using reverse engineering technique. Brackets models were constructed using stainless steel MBT prescription of slot size 0.022 x 0.028-in. Stainless steel archwire of 0.019 x 0.025-in dimension and a nickel-titanium closed coil spring was fabricated and attached from crimpable hook to the mini-implant head, generating retraction force of 150g (Fig 3).The mini-implants were inserted at angulations of 60° and 90° to the buccal surface of alveolus. Three FEM models were generated, in which all the parameters were kept the same, except the insertion angulation of the mini-implant. Material properties assigned to the FEM were tabulated in Table 2.^9^

**Table 2:** Material property data representation (10).

Material	Elastic modulus E (GPa)	Poisson’s ratio
Tooth	20.7	0.30
Compact bone	14.7	0.30
Cancellous bone	1.5	0.30
Titanium mini-implant	114	0.34
Bracket and wire	179	0.30
Nickel-titanium	36	0.33

**Table 3:** Nodes and elements defined for each model.

	Angulation			
	90°		60°	
Mini-implants	Nodes	Elements	Nodes	Elements
M1	357,580	1,529,563	357,737	1,529,610
M2	357,822	1,531,187	357,714	1,531,233
M3	357,103	1,527,129	357,214	1,527,246

#### Statistical analysis

Descriptive statistics included calculation of mean and standard deviation for TSF and pull-out tests of three different mini-implants. Shapiro-Wilk’s normality test was used to verify the equality of variance. One-way ANOVA and Tukey *post-hoc* tests were used to compare the TSF and pull-out strength of the mini-implants, within as well as between the groups. The level of significance was *p*<0.05. Data were analyzed using SPSS software v. 23.0.

## RESULTS

### SEM and pull-out test

Since the Shapiro-Wilk’s normality test confirmed the equality of variance, one-way ANOVA was used for the between-group comparisons. The mean thread depth, pitch and TSF of M1 was found to be 0.088mm, 0.426mm and 20.667%, respectively; for M2, it was 0.217mm, 0.849mm and 25.483%, respectively; and for M3, it was 0.097mm, 0.507mm and 19.100% respectively. ANOVA showed statistically significant difference for thread depth and pitch for all the three mini-implants, and statistically insignificant for TSF (Table 4). *Post-hoc* Tukey test showed: statistically significant difference for thread depth between M1 and M2, and M2 and M3; not significant difference between M1 and M3; and statistically significant difference between all the groups for the pitch of the mini-implants (Table 5).

**Table 4:** Comparisons of mean depth, pitch, TSF and peak load among all three groups, by analysis of variance.

Parameter	Orthoimplant (M1)		Tomas (M2)		Vector TAS (M3)		
	Mean	SD	Mean	SD	Mean	SD	ANOVA p value
Depth (mm)	0.088	0.019	0.217	0.046	0.097	0.027	<0.001*
Pitch (mm)	0.088	0.049	0.849	0.024	0.507	0.010	
TSF (%)	20.667	4.894	25.483	4.967	19.100	5.277	0.107
Peak load (kN)	0.181	0.018	0.142	0.030	0.138	0.025	0.017*

* p < 0.05.

**Table 5:** Multiple comparisons between groups by Tukey post-hoc test.

Parameter	M1-M2		M1-M3		M2-M3	
	Mean difference	p value	Mean difference	p value	Mean difference	p value
Depth (mm)	-0.130	<0.001*	-0.010	0.871	0.120	<0.001*
Pitch (mm)	-0.423	<0.001*	-0.080	0.002*	0.343	<0.001*
TSF (%)	-4.817	0.255	1.567	0.854	6.383	0.105
Peak load (kN)	0.039	0.040*	0.043	0.024*	0.004	0.962

* p < 0.05.

The mean values of M1, M2 and M3 for the pull-out test were 0.181kN; 0.142kN and 0.138kN, respectively. Differences were statistically significant (Table 4). 

### Finite element method

The results showed changes in terms of von Mises stress and principal stresses. The magnitude of stresses developed in reaction to applied retraction force is mentioned in Table 6 and the pattern of stress distribution is described below.

**Table 6:** Magnitude of stresses developed under same load and different mini-implant angulations.

Mini-implant	Mini-implant				Cortical bone			
	Insertion angle							
	90 degree		60 degree		90 degree		60 degree	
	Maximum (MPa	Minimum (MPa)	Maximum (MPa	Minimum (MPa)	Maximum (MPa	Minimum (MPa)	Maximum (MPa	Minimum (MPa)
M1	23.72	0.1056	28.01	0.062	2.4184	0.078	2.9524	0.077
M2	80.03	0.1382	107.06	0.014	6.7626	0.134	5.4152	0.115
M3	17.01	0.0625	14.89	0.008	3.8516	0.081	3.6095	0.071

### Mini-implant

**Figure 4:**
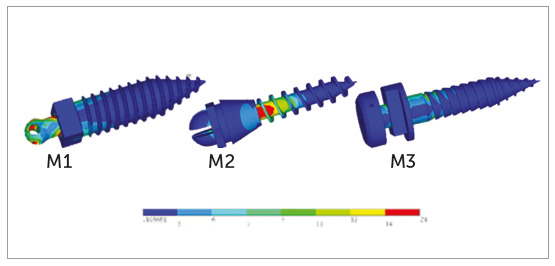
M1, M2, M3: Pattern of stress distribution along mini-implant length at 90° insertion angulation.

For M1 at 90° insertion angle, maximum stress was observed on the head of the mini-implant at the point of attachment with the retraction spring and at the junction of the head and transmucosal collar (neck). The stresses gradually decreased from first thread until fourth thread. Minimum levels of stress remained constant throughout the length of the mini-implant (Fig 4, M1).

**Figure 5:**
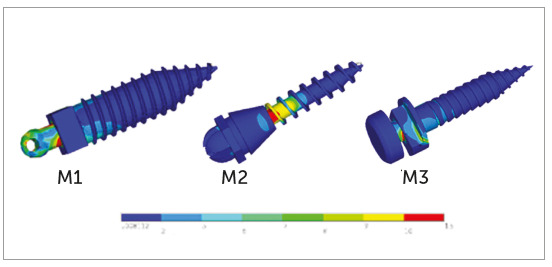
M1, M2, M3: Pattern of stress distribution along mini-implant length at 60° insertion angulation.

At 60° insertion angle, a small portion of maximum stress was observed at the junction of the head and neck. The stresses gradually decreased from first thread until fourth thread. Minimum levels of stress remained constant throughout the length of the mini-implant. The maximum von Mises stresses at 90° and 60° insertion angle were 23.72 MPa and 29.01 MPa, respectively (Fig 5, M1).

For M2 at 90° insertion angle, maximum stress was observed in the first and second threads. Stresses decreased towards the neck and below the third thread. The stresses gradually decreased from fourth and fifth thread. The stresses remained minimal from fifth thread to the tip of the mini-implant (Fig 4, M2).

At 60° insertion angle, maximum stresses were observed at the larger portion of first and second threads, and a smaller portion of third thread and neck of the mini-implant. Stresses were decreased to a small portion of fourth thread, and the stresses reached minimum levels and remained constant at the head and a larger portion of neck and from fifth thread to the tip of the mini-implant. The maximum von Mises stresses at 90° and 60° insertion angle were 80.03 MPa and 107.06 MPa, respectively (Fig 5, M2).

For M3 at 90° insertion angle, maximum stress was observed at the first and second threads and a small portion of the head, at the point of attachment of retraction spring. The stresses gradually reduced at the neck and a small portion of third and fourth threads, and from there the stresses reached minimum level and remained constant throughout the length of the mini-implant (Fig 4, M3).

At 60° insertion angle, maximum stresses were observed at the junction of head and neck. The stresses reduced from the threaded body and at the small portion of third thread. The stresses reached a minimum level and remained constant throughout the length of the mini-implant. The maximum von Mises stresses at 90° and 60° insertion angle were 17.01 MPa and 14.89 MPa, respectively (Fig 5, M3).

### Cortical bone

**Figure 6:**
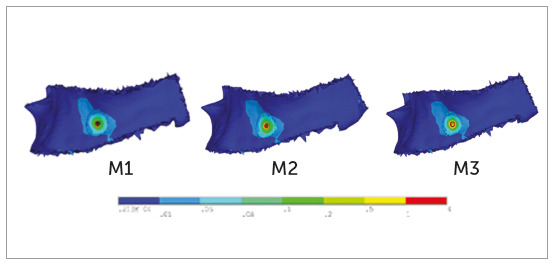
M1, M2, M3: Pattern of stress distribution in cortical bone at 90° insertion angulation.

**Figure 7:**
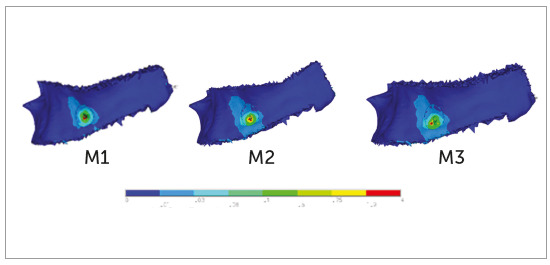
M1, M2, M3: Pattern of stress distribution in cortical bone at 60° insertion angulation.

For M1 at the 90° and 60° insertion angle, the pattern of stress distribution was the same, where maximum stresses were observed at the mesial, distal and apical to the mini-implant. Stresses uniformly decreased in the form of concentric circles as it is moved away from the mini-implant and reached closer to the upper small portion of the lower crest of the cortical bone (Figs 6 and 7, M1).

For M2 and M3 at the 90° insertion angle, the pattern of stress distribution was similar to what was observed for the M1. At 60° insertion angle, the stresses at M2 and M3 were close to M1, with a main difference that the stresses reached the broader area of lower crest of the cortical bone (Figs 6 and 7, M2-M3).

## DISCUSSION

### SEM and pull-out test

The fundamental parameter for primary retention of TADs is the pull-out strength, which is linked to bone related factors^11-12^ and mini-implant design factors like diameter, pitch, thread depth and TSF.^2^ ^,^ ^13^ The TSF and relative pull-out strength values in the current study were: 20.6%, 0.181kN for M1; 25.4%, 0.142 kN for M2; and 19.1 %; 0.138kN for M3.

The results of this study showed no correlation between TSF and pull-out strength. Previous literature has shown contradicting conclusions. Radwan et al^5^ concluded that decreased TSF led to increased pull-out forces and thus, to higher primary stability; however, Migliorati et al^2^ ^,^ ^13^ reported that a larger TSF provided higher primary stability.

The results in the current study showed a non-significant difference in TSF and significant difference in pull-out force between the three groups. M1 had the highest value of pull-out force, followed by M2 and, finally, M3 (Table 4). The results of the current study indicate that different geometric design parameters like pitch, thread depth and diameter of mini-implant, apart from TSF, influenced the mechanical stability of the mini-implant.

The results of the current study showed no definitive correlation between the pitch and pull-out values, as shown in Table 4. Brinley et al^14^ reported that a decrease in pitch led to increase in pull-out force and therefore higher primary stability. In contrast, Migliorati et al^2^ reported that there was a positive correlation between pitch and pull-out force when mini-implants of less than 1mm pitch were inserted in cortical thickness of 2.2mm. The reason for increased primary stability in that study could be due to more thread engagement in cortex in mini-implants with < 1 mm pitch when cortical bone was 1.0 - 2.0mm in width.

The current study showed no definitive correlation between the thread depth and pull-out values, as shown in Table 4. Chang et al^4^ concluded that pull-out resistance decreased abruptly as the thread depth increased from 0.32 to 0.40 mm. In the present study, the thread depth is within 0.32mm for all the three mini-implants. Mini-implants used in this study have three different diameters (1.8mm, M1; 1.6mm, M2; and 1.4mm, M3). The results of this study showed a definitive correlation between diameter and pull-out force (Table 4).

The greater the diameter of the mini-implant, the greater the bone compression is, leading to an increased primary stability.^15^ Results of this study were in agreement with results reported in previous studies^16^ ^,^ ^17^ ^,^ ^18^ Walter et al^18^ stated that mini- implants with <1.2 mm in diameter should be avoided to prevent failure. Studies on fracture resistance have related the relationship between diameter and strength; they considered that a 0.1 mm increase in core diameter should give greater fracture resistance.^19^ OrthoImplant implants can safely resist the high levels of orthodontic forces used for *en-masse* teeth retraction and molar uprighting.

#### FEM study

The insertion angle of mini-implant varies most often according to clinical preference. Therefore, it is necessary to compare the efficacy in terms of stress induced in the metal and bone among mini-implants of various design and insertion angle with orthodontic loading.^20^

#### Stress analysis on the mini-implant and cortical bone

In the present study, it was observed that for a given load, i.e. 150g, the stress values on mini-implant and in surrounding bone were higher for M2 with 60° and 90° insertion angle, followed by M1 and M3, respectively (Table 6). M2 mini-implant, which has a greater thread depth and smaller taper design, showed higher stresses when compared to the other two mini-implants. The results of the present study are in agreement with Chang et al,^4^ who concluded that mini-implant with greater thread depth, smaller taper and short taper length generated higher stresses on the bone and thread elements in lateral loading condition.

The stress levels in the mini-implant increased with reduction in the insertion angle for M2 and M1. The results are in agreement with studies by Woodall et al,^21^ and Lee et al,^22^ who concluded that placing mini-implant at 90° insertion angle increases the biomechanical stability of mini-implant. The authors also stated that oblique/acute angulations potentially creates longer lever arms, making the threads not completely engaged into the bone, creating increased stress and displacement around the mini-implant, negatively contributing to the primary stability.^15^ ^,^ ^23^ In the present study, the stress levels in M3 increased with an increase in the insertion angle, and the reason could be the reduced diameter of the mini-implant.

For M2 mini-implant, high stresses were distributed on the uppermost threads at the neck of the mini-implant near the margin of bone, with both insertion angulations. High stresses were observed on the head of mini-implant at the point of attachment of the retraction spring, with respect to M1 and M3 with both insertion angulations. This pattern of stress distribution on mini- implant (M1 and M3) was in agreement with studies conducted by Ammar et al^24^ and Gracco at al.^6^ Benedict et al^25^ and Ammar et al^24^ in their studies suggested that 2-3mm of the implant’s endo-osseous length is most critical in terms of stress response under tangential loading, and the results of the present study were in agreement with that. Mini-implants manufacturers should expect more failures at top three threads.

However, the stress values in the current study were below the yield stress of titanium (692 Mpa),^26^ thus indicating that all mini-screws have sufficient strength to resist forces during orthodontic loading.

Highest amount of principal stress in the bone were seen with the M2 mini-implant and the least amount of principal stress were seen for the M1 type. The results were in agreement with previous studies^27^ ^-^ ^28^ that concluded that mini-implants with smaller pitch showed less stress within the bone. In the present study, M1 and M3 had smaller pitch when compared to M2, so smaller amount of stresses was observed with M1 and M3 mini-implants.

The maximum principal stress in bone for both insertion angles indicated that the stress decreased from 60° to 90° for M1, but this decrease in stress distribution was observed to be marginal. These findings were in agreement with previous studies,^8^ ^,^ ^29^ which reported that when the mini-implant insertion angle was increased from 60° to 90°, the stress in the surrounding bone decreased. However, for M2 and M3 there was a marginal increase in stress distribution from 60° to 90° insertion angle. The maximum stress value of 6.7626 MPa was seen with 150-g load and at 90° insertion angulation. As this value is way smaller compared to the 133 MPa yield stress of cortical bone, it can be inferred that no significant adverse changes will be seen in cortical bone.

## CONCLUSION

» Within the limitation of this study involving the finite element analyses and mechanical testing of different mini-implants, the result demonstrated that Orthoimplant type with a larger diameter, smaller pitch and shorter taper length have better primary stability, and also have low stresses within the mini-implants and surrounding bone amongst the three groups.
» The favorable insertion angulation found was 90°, as it provides better primary stability and low stresses in the mini-implant and surrounding bone under orthodontic loading.
» Further research is required for optimization of thread-parameters and its validation on living bone tissue.
